# Circulating Cytokines in Metastatic Breast Cancer Patients Select Different Prognostic Groups and Patients Who Might Benefit from Treatment beyond Progression

**DOI:** 10.3390/vaccines10010078

**Published:** 2022-01-05

**Authors:** Matteo Paccagnella, Andrea Abbona, Andrea Michelotti, Elena Geuna, Fiorella Ruatta, Elisabetta Landucci, Nerina Denaro, Paola Vanella, Cristiana Lo Nigro, Danilo Galizia, Marco Merlano, Ornella Garrone

**Affiliations:** 1 Translational Oncology ARCO Foundation, 12100 Cuneo, Italy; abbona.andrea@gmail.com; 2 Department of Medical Oncology, Azienda Ospedaliero Universitaria Pisana, 56126 Pisa, Italy; andre.michelotti@gmail.com (A.M.); ely.landucci@gmail.com (E.L.); 3 Multidisciplinary Oncology Outpatient Clinic, Candiolo Cancer Institute FPO-IRCCS, 10060 Candiolo, Italy; elena.geuna@ircc.it (E.G.); danilo.galizia@ircc.it (D.G.); 4 Department of Medical Oncology, S. Croce e Carle Teaching Hospital, 12100 Cuneo, Italy; fiorella.ruatta@gmail.com (F.R.); nerinadenaro@gmail.com (N.D.); paola.vanella@libero.it (P.V.); 5 Laboratori Centrali, EO Ospedali Galliera, 16128 Genova, Italy; c_lo_nigro@hotmail.com; 6 Experimental Cell Therapy Lab, Candiolo Cancer Institute, FPO-IRCCS Candiolo, 10060 Torino, Italy; marcocarlo.merlano@ircc.it; 7 Department of Medical Oncology, Fondazione IRCCS Ca’ Granda Ospedale Policlinico Maggiore, 20122 Milano, Italy; ornella.garrone@policlinico.mi.it

**Keywords:** eribulin, metastatic breast cancer, cytokines, treatment beyond progression

## Abstract

Cancer induces immune suppression to overcome its recognition and eradication by the immune system. Cytokines are messengers able to modulate immune response or suppression. There is great interest in the evaluation of their changes during treatment in order to identify their relationship with clinical outcome. We evaluated 18 cytokines in breast cancer patients treated with eribulin before starting treatment (T0) and after four courses of therapy (T1). Longitudinal modifications were considered and cytokine clusters through PCA and HCPC correlated to patients’ outcomes were identified. Forty-one metastatic breast cancer patients and fifteen healthy volunteers were included. After clustering, we identified at T0 six patient clusters with different risk of relapse and death. At T1, only four clusters were identified, and three of them accounted for thirty-eight of forty-one patients, suggesting a possible role of treatment in reducing heterogeneity. The cluster with the best survival at T1 was characterized by low levels of IL-4, IL-6, IL-8, IL-10, CCL-2, CCL-4, and TGF-β. The cluster showing the worst survival encompassed high levels of IL-4, IL-6, IL-8, IL-10, CCL-2, and IFN-γ. A subgroup of patients with short progression-free survival (PFS) and long overall survival (OS) was comprised in the cluster characterized by low levels of CCL-2, IL-6, IL-8, IL-10, and IL-12 at T0. Our data support the prognostic significance of longitudinal serum cytokine analysis. This approach may help identify patients for whom early treatment stop avoids needless toxicity or might justify treatment beyond early progression. Further investigations are required to validate this hypothesis.

## 1. Introduction

The tumor microenvironment (TME) drives the dynamic interaction between tumor and immune cells, largely mediated by cytokines and chemokines that exert their biological actions even distantly moving through blood circulation, for instance supporting immune-cell recruitment and cancer metastatization [1].

Chemotherapy has the capability to preferentially kill cancer cells, however it may stimulate anti-tumor immune response. The immunomodulatory effect of chemotherapy reflects its ability to induce the release of antigens from dying cancer cells (i.e., immunogenic cell death, ICD) or to induce off-target effects involving immune cells and the release of cytokines and chemokines. The two effects are not mutually exclusive: for example, anthracyclines induce ICD while upregulating CXCL10, IL-6, type I and II interferons, and other cytokines [2,3,4].

Eribulin (Halaven^®^) is among the newest chemotherapy agents available in clinical practice. The drug is approved by European Medical Agency (EMA) and Food and Drug Administration (FDA) for the treatment of metastatic breast cancer (mBC) patients after at least one or two previous lines of therapy, respectively [5,6].

Eribulin inhibits microtubules growth, leading to non-productive tubulin aggregates preventing the formation of the mitotic spindle, which in turn induces irreversible mitotic block and apoptosis, as reviewed previously [7].

Along with the classical mechanism of action, eribulin exerts also important ‘off-target’ immune effects, making this drug particularly interesting. Indeed, eribulin modulates many cytokines [8] and, in particular, reduces tumor growth-factor-β (TGF-β) in both experimental and human models [9,10], and induces vascular remodeling leading to a better perfusion of the tumor mass [11].

We demonstrated that, in vitro, eribulin effectively counteracts endothelial mesenchymal transition, driven by TGF-β, indicating a possible effect on vascular remodeling. In addition, it is able to upregulate the endothelial adhesion protein ICAM-1, suggesting that the drug may facilitate T-cell homing [12].

Finally, in mBC patients treated with eribulin, a significant decrease of TGF-β leads to a better outcome [13].

However, focusing on a single cytokine is insufficient as far as they interact each other within a complex network [14,15]. Thus, the final effect on the TME, and possibly on the treatment activity, correlates with their dynamic interplay and is context dependent [16].

Indeed, other authors have already profiled large panels of circulating cytokines in breast cancer patients. Jabeen et al. profiled 27 circulating cytokines of 159 breast cancer patients and characterized their tumor microenvironment [17]. Kawaguchi et al. [18] profiled 26 circulating cytokines in 185 patients and 54 healthy volunteers and identified specific cytokine signature of metastatic and non-metastatic patients with a mathematical model to distinguish healthy volunteers from patients. However, few studies have examined the longitudinal changes of circulating cytokines during treatment.

In the present study, we investigated the change of 18 cytokines in breast cancer patients during treatment with eribulin. Our aim was to evaluate cytokine modification during treatment and identify clusters correlated to patients’ outcomes.

## 2. Materials and Methods

Patients were treated with eribulin following EMA criteria. Blood samples for cytokine analysis were obtained at baseline (T0) and after four cycles of chemotherapy (T1), corresponding to the first-response evaluation.

We retrospectively grouped patient population into four groups based on median progression-free survival (PFS) and median overall survival (OS) of the whole population:

Group 1. Patients with both PFS and OS below the median;
Group 2. Patients with PFS below the median and OS above the median;
Group 3. Patients with PFS above the median and OS below the median;
Group 4. Patients with both PFS and OS above the median.

Finally, we collected blood samples also from a group of healthy volunteers (HV).

### 2.1. Plasma Collection

For each patient, 12 milliliters of peripheral blood samples were stored in EDTA-treated Vacutainer (BD, Franklin Lakes, NJ, USA). Plasma samples were obtained by centrifugation step at 340× *g* for 10 min at room temperature (RT) and promptly stored at −80 °C until use.

### 2.2. Cytokines

We selected cytokines on the basis of their Th1 (pro-inflammatory, anti-tumor) or Th2 (anti-inflammatory, pro-tumor) effects, their association with survival, or recognized immunosuppressive effects. IL-2, IL-12, CXCL-10 TNF-α, and IFN-γ are considered Th1 cytokines, while IL-4, IL-5, IL-10, IL-13, and CCL-22 are considered Th2 cytokines [19]. High levels of IL-6 and IL-8 are associated with poor survival in many tumors [20,21], and TGF-β and VEGF are both major immunosuppressive factors [22,23].

### 2.3. Cytokine Measurement

Concentrations of all cytokines but IL-21 were determined using the Ella Simple Plex system (ProteinSimple™, San Jose, CA, USA) according to the manufacturer’s instructions.

IL-21 was assessed with ELISA method (R&D System Minneapolis, MN, USA).

Briefly, as previously described [13], a twofold dilution of each plasma sample was spun for 15 min at 1000× *g* and added to the Simple Plex cartridge. The cartridge was then inserted into the reactor and run for 90 min at RT. Concentrations were expressed as pg/mL.

All blood samples were tested centrally at the Translational Research Laboratory ARCO Foundation at S. Croce and Carle Teaching Hospital in Cuneo, Italy, and assessed in duplicate. The average of each duplicate was considered at each point.

### 2.4. Statistical Analyses

The exploratory nature of this translational study did not allow *a priori* sample size and statistical power calculation, therefore the sample size of the patient population and of the healthy volunteers was arbitrary established.

Differences in the median cytokine values were analyzed using a non-parametric Mann–Whitney U test and Wilcoxon signed-rank test for paired samples. Principal Component Analysis (PCA) and Hierarchical Clustering on Principal Components (HCPC) were performed on cluster subjects at T0 and T1 using circulating-cytokine concentrations previously normalized in z-score.

PFS and OS were estimated using Kaplan–Meyer method and relative hazard ratio (HR) was performed by the Cox model.

PFS was defined as the time elapsed between the start of eribulin and progressive disease or death from any cause, whichever occurred first, or at the date of last follow-up for censored patients.

OS was defined as the time elapsed between the start of eribulin and death from any cause or the date of the time of the last follow-up for censored patients.

The Mann–Whitney U test and Wilcoxon signed-rank test were performed with GraphPad v.5. Kaplan–Meyer and the Cox model were performed with SPSS V.24. PCA and HCPC were computed with R v.3.5.3 by the FactoMiner R package.

In all tests, a *p* value equal or lower to 0.05 was regarded as significant. Bonferroni’s correction was applied for the multiplicity test [24]. If not specified, a *p*-value is considered NS (not significant).

## 3. Results

### 3.1. Patient Population

We included 41 mBC patients. Median age was 62; median Eastern Cooperative Oncology Group (ECOG) performance status (PS) was 0; among patients, 83% and 7% had hormone receptor-positive and HER2-positive disease, respectively. Six patients (14.6%) had triple-negative disease. Twenty-nine patients (70.7%) had liver metastasis. Main patients’ characteristics are reported in Table 1.

### 3.2. Healthy Volunteers

Blood samples were collected from 15 HV. Median age was 47 years (range 28–65), with two males and thirteen females.

### 3.3. Treatment Effects

Partial response (PR) was recorded in 10 patients (24.4%), and six patients (14.6%) achieved stable disease (SD), lasting 6 months or more, with clinical benefit rate of 39%.

According to the grouping system described above, we divided our population as follows:

Group 1 (patients with both PFS and OS below the median): 12 patients (29.2%), median PFS 2.8 months (range 2.5–3.6), and median OS 4.6 months (range 3.0–10.8).
Group 2 (patients with PFS below the median and OS above the median): 9 patients (21.9%), median PFS 2.8 months (range 2.7–3.5), and median OS 15 months (range 11.2–23.8).
Group 3 (patients with PFS above the median and OS below the median): 9 patients (21.9%), median PFS 5.3 months (range 3.7–7.6), and median OS 7.9 months (range 5.3–9.8).
Group 4 (patients with both PFS and OS above the median): 11 patients (26.8%), median PFS 9.9 months (range 4.5–24.4), and median OS 19.6 months (range 11.7–33.7).

Due to the apparent contrasting results emerging from the analysis of the patients in group 2 (short PFS and long OS), we focused our attention on this cohort.

Detailed characteristics of patients allocated in group 2 are reported in Table 2.

In particular, all patients in group 2 received further treatment after eribulin, but no one obtained treatment response.

### 3.4. Cytokine Profile in 15 HV and in 41 Patients

We determined the median values of the 18 cytokines in 15 HV and their values were compared to the values observed in 41 patients (Table 3).

We analyzed differences between HV and patients at T0.

We found that, at T0, patients had higher values of IL-6, IL-8, IL-10, IL-21, TGF-β, VEGF, IFN-γ, CCL-2, CCL-4, and CXCL-10 and lower levels of IL-4 and IL-13 compared to HV.

At T1, after four courses of eribulin, patients maintained higher levels of IL-6, IL-8, IL-10, IL-21, TGF-β, VEGF, IFN-γ, CCL-2, CCL-4, and CXCL-10 whilst IL-4 switched from significantly lower to significantly higher value compared to HV.

**Figure 1:**
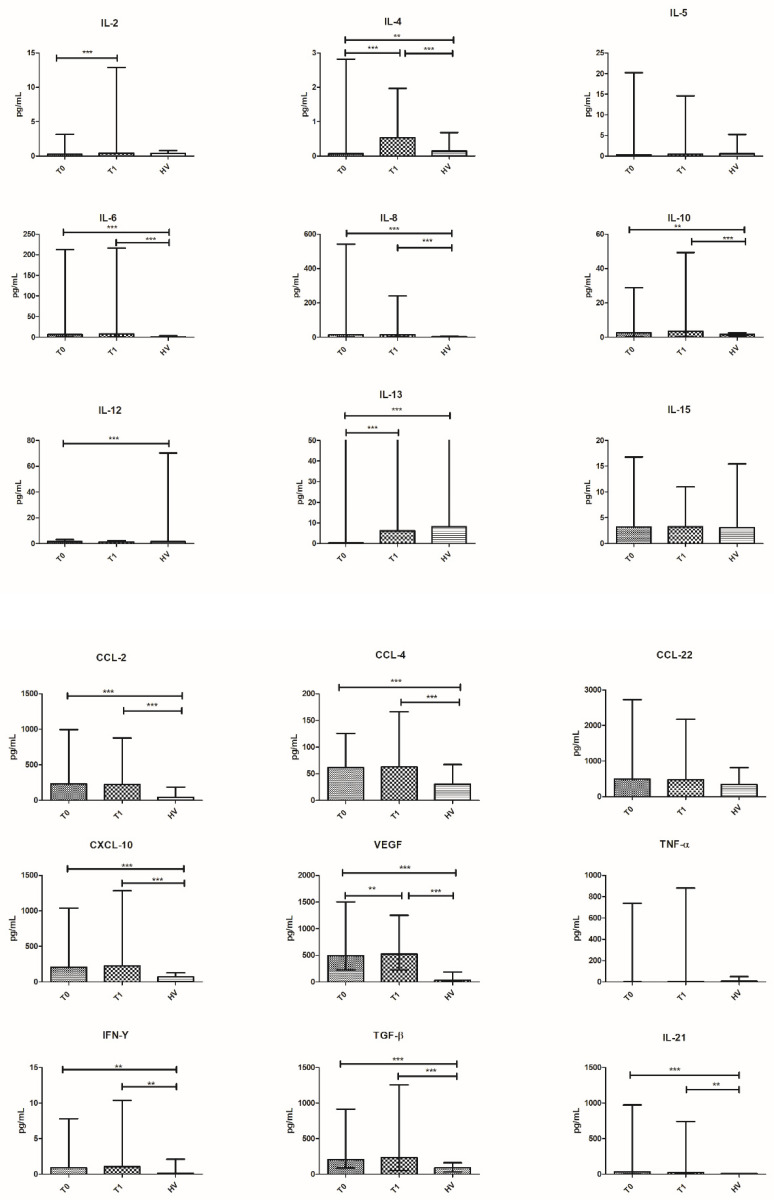
Cytokine distribution at T0 and T1 in all patients and HV. Plasma concentration is expressed in pg/mL and plotted on the *y*-axis; time points are on the *x*-axis. Data are expressed as median with range. *** *p* < 0.001, ** *p* < 0.01, * *p* < 0.05. CCL, C-C motif ligand; IL, interleukin.

Then, we compared the concentration of the cytokines of patients at timepoints T0 and T1. A significant increase in of IL-2 (*p* = 0.0004), IL-4 (*p* < 0.0001), IL-5 (*p* = 0.023), IL-13 (*p* = 0.0001), and VEGF (*p* = 0.0045) was found from T0 to T1 (Table 3 and Figure 1).

### 3.5. Patients’ Clusterization and Their Cytokine Profile at T0

The concentration of the 18 cytokines recorded before starting eribulin (T0) was used to cluster patients through PCA and HCPC methods.

**Figure 2:**
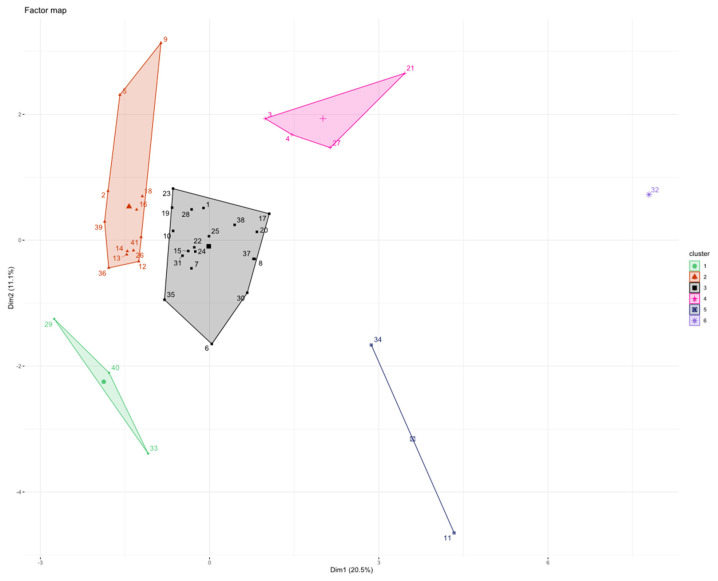
HCPC clustering with all cytokine values normalized at T0. On the *x*-axis is plotted first PC and on the *y*-axis second PC. Each patient is represented by a single dot number, labelled with a color of the cluster in which it belongs. Centroids are shown as bigger symbol with no number.

Using HCPC at T0, we found that patients were distributed in six clusters (C): C1^T0^ (*n* = 3), C2^T0^ (*n* = 12), C3^T0^ (*n* = 19), C4^T0^ (*n* = 4), C5^T0^ (*n* = 2), C6^T0^ (*n* = 1) (Figure 2).

Patients in HCPC at T0 were allocated as follows:

- C1^T0^ = 3 patients (1 patient from group 3 and 2 patients from group 4);
- C2^T0^ = 12 patients (1 patient from group 1, 7 patients from group 2, and 4 patients from group 4);
- C3^T0^ = 19 patients (8 patients from group 1, 1 patient from group 2, 6 patients from group 3, and 4 patients from group 4);
- C4^T0^ = 4 patients (2 patients from group 1, 1 patient from group 2, and 1 patient from group 3);
- C5^T0^ = 2 patients (1 patient from group 1 and 1 patient from group 3);
- C6^T0^ = 1 patient from group 4.

Although many patients gathered in C2^T0^ and C3^T0^, we observed that the population was dispersed among many clusters.

Patients in C2^T0^, mainly belonging to group 2, had significantly better OS compared to patients in C3^T0^ (median OS 18.4 months and 8.9 months, respectively). Indeed, patients in C2^T0^ had a significant mortality-risk reduction compared to all patients taken together (HR = 0.35, 95% C.I. 0.16 to 0.78, *p* = 0.01).

Moreover, we performed Cox multivariate analysis in order to evaluate the impact of previous lines of treatment received before eribulin (discriminating patients treated in the second or more-advanced lines), the metastatic site (visceral vs. bone/soft tissue), and the number of metastatic sites in patients at C2^T0^. It seems that belonging to C2^T0^ is an independent prognostic factor as it is the only one that remains significant. HR = 0.32 and *p* = 0.01 (Supplementary Materials: Figure S1).

When comparing patients in C2^T0^ with those in C3^T0^, patients in the former cluster were characterized by significant lower levels of CCL-2, IL-6, IL-8, IL-10, and IL-12 at baseline.

Patients in C2^T0^ had lower values of IL-6, IL-10, CCL-2, and CXCL-10 compared to those in C4^T0^.

HV had lower levels of IL-6, IL-8, CCL-2, CXCL-10, and VEGF compared to patients in C1^T0^, C2^T0^, and C3^T0^.

HV had lower levels of IL-10 and CCL-4 compared to patients in C3^T0^ and C4^T0^; TGF-β level was lower in HV compared to patients in C2^T0^ and C3^T0^ and also IL-21 level was lower compared to patients in C3^T0^.

**Figure 3:**
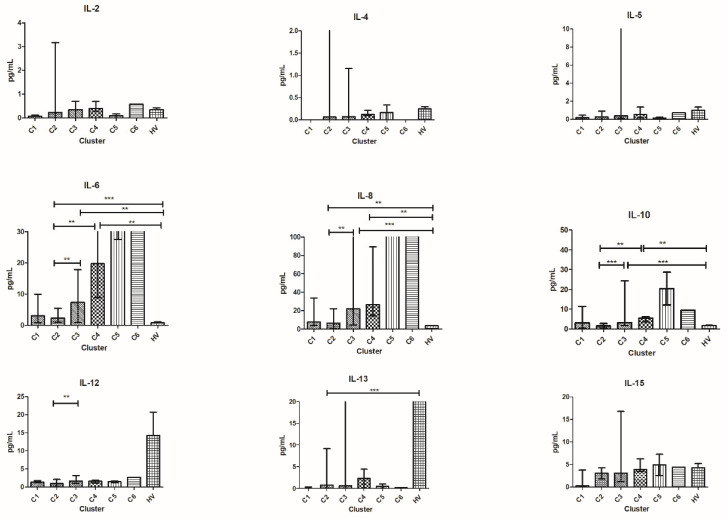

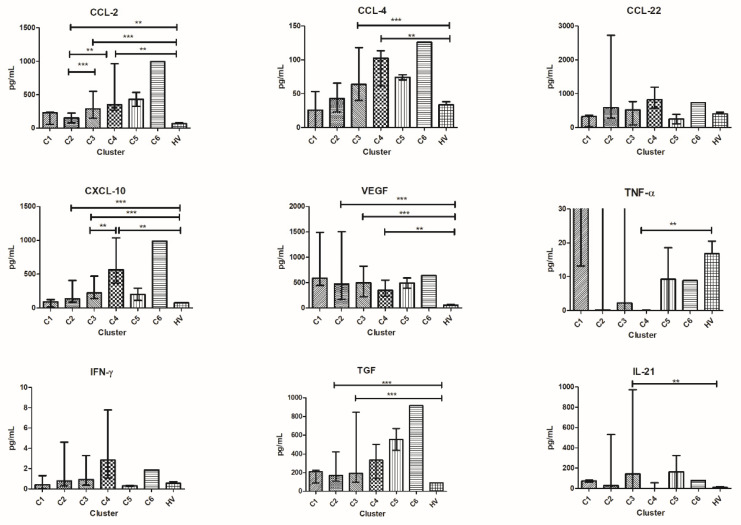
Cytokine distribution at T0 in the six clusters and HV. Plasma concentration is expressed in pg/mL and plotted on the *y*-axis; clusters of patients are on the *x*-axis. For TNF-α, IL-4, IL-5, IL-6, IL-8, and IL-13 outliers are not shown for graphical choice. Data are expressed as median with range. *** *p* < 0.001, ** *p* < 0.01, * *p* < 0.05. CCL, C-C motif ligand; IL, interleukin.

In addition, HV had higher levels of IL-13 compared to patients in C2^T0^ and higher levels of TNF-α compared to patients in C4^T0^ (Figure 3).

**Figure 4:**
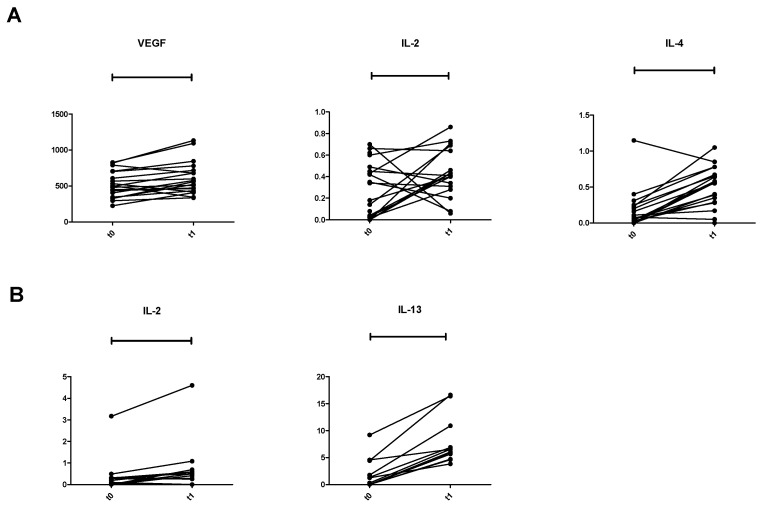
Longitudinal cytokine variation. (**A**) Spaghetti plot of 19 patients included in C3 from T0 to T1. (**B**) Spaghetti plot of 12 patients included in C2 from T0 to T1. IL, interleukin.

We investigated the longitudinal shifting of the cytokines from T0 to T1 of patients included in the clusters identified at T0 and we found that patients in C3^T0^ had a significant increase in IL-2, IL-4, and VEGF (*p* = 0.034, *p* = 0.0006, and *p* = 0.01, respectively), while patients in C2^T0^ showed a significant longitudinal increase of IL-2 and IL-13 from the two time-points (*p* = 0.02 and *p* = 0.0005, respectively) (Figure 4A,B).

No significant changes were observed among the other clusters. It must be stressed that the remaining clusters, all together, accounted for 10 patients only.

### 3.6. Patients’ Clusterization and Their Cytokine Profile at T1

**Figure 5:**
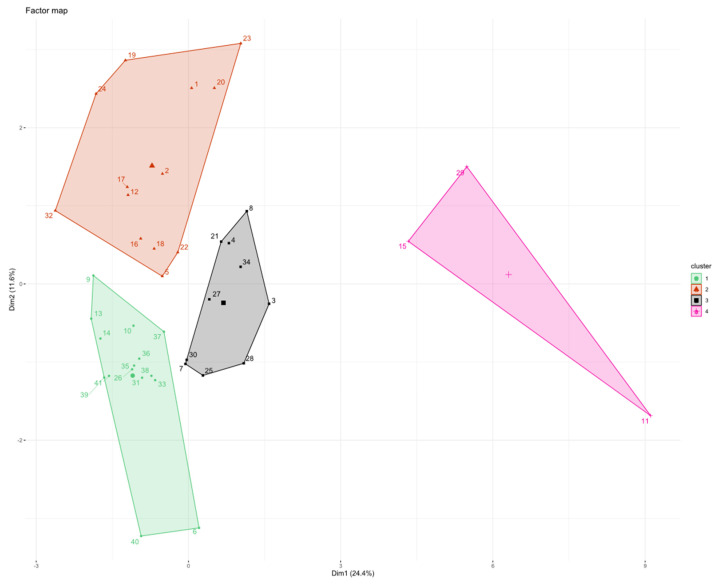
HCPC clustering with all cytokine values normalized at T1. On the *x*-axis is plotted first PC and on the *y*-axis second PC. Each patient is represented by a single dot number, labelled with a color of the cluster in which it belongs. Centroids are shown as bigger symbol and with no number.

After treatment with eribulin (T1), PCA and HCPC grouped patients into four clusters: C1^T1^ (*n* = 15), C2^T1^ (*n* = 13), C3^T1^ (*n* = 10), and C4^T1^ (*n* = 3) (Figure 5).

Patients in HCPC at T1 were distributed as follow:

- C1^T1^ = 15 patients (1 patient from group 1, 4 patients from group 2, 3 patients from group 3, and 7 patients from group 4);
- C2^T1^ = 13 patients (4 patients from group 1, 4 patients from group 2, 3 patients from group 3, and 2 patients from group 4);
- C3^T1^ = 10 patients (5 patients from group 1, 1 patient from group 2, 2 patients from group 3, and 2 patients from group 4);
- C4^T1^ = 3 patients (2 patients from group 1 and 1 patient from group 3).

**Figure 6:**
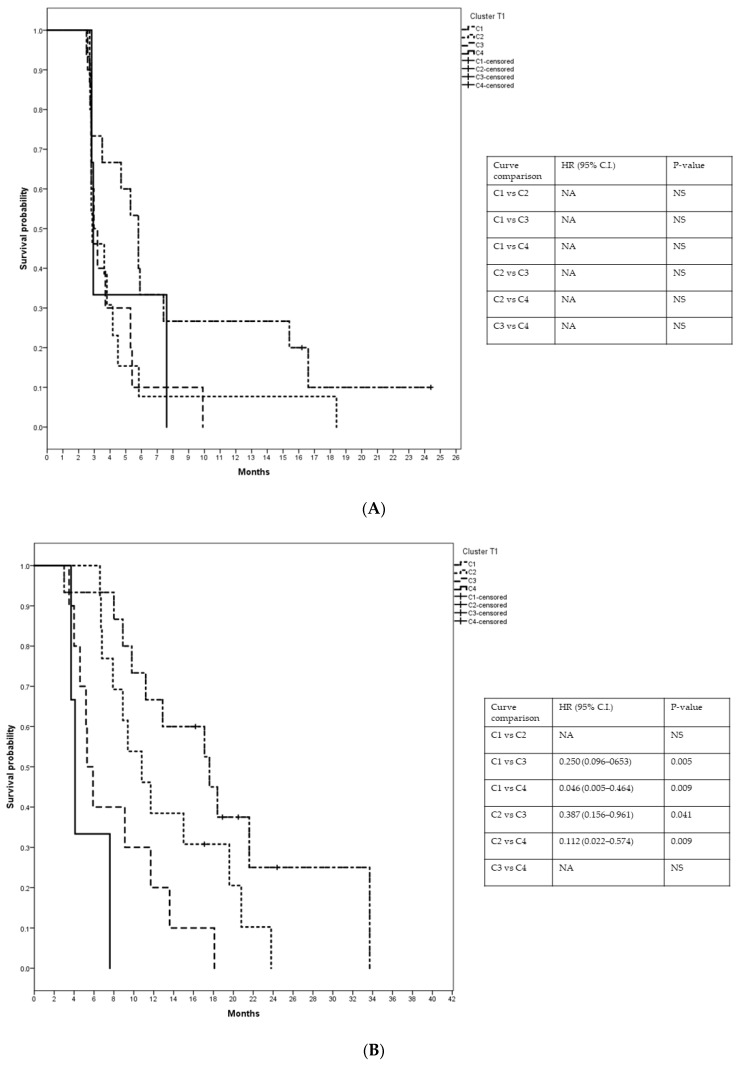
Kaplan–Meyer curves illustrate (**A**) PFS and (**B**) OS of four clusters at T1. NA = not available, NS = not significant.

Patients in C1^T1^ had median PFS and OS of 5.8 months (C.I.: 4.44–7.16) and 17.6 months, (C.I.: 9.49–25.71), respectively; in C2^T1^, median PFS and OS were 2.8 months (C.I.: 2.34–3.40) and 10.8 months, (C.I.: 7.51–14.09), respectively; in C3^T1^, median PFS and OS were 2.97 months (C.I.: 2.35–3.59) and 5.3 months (C.I.: 4.22–6.39), respectively, and in C4^T1^, median PFS and OS were 2.93 months (C.I.: 2.77–3.09) and 4.1 months (C.I.: 3.46–4.74), respectively (Figure 6).

C1^T1^ included patients with better median PFS and OS compared to the other clusters together. In C1^T1^, median PFS was 5.8 months compared to 2.9 months, and OS 17.6 months compared to 7.9 months. Cox analysis performed for PFS and OS indicated a significant risk reduction favoring C1^T1^ (HR = 0.49; 95% C.I. 0.25–0.98, *p* = 0.046; HR = 0.37; 95% C.I. 0.17–0.78, *p* = 0.009 for PFS and OS, respectively).

Comparing cytokine values among clusters, patients in C1^T1^ had lower median values of TGF-β, CCL-4, and CCL-22 compared to patients in C2^T1^.

Patients in C1^T1^ had lower median values of IL-4, IL-6, and CCL-4 compared to those in C3^T1^.

**Figure 7:**
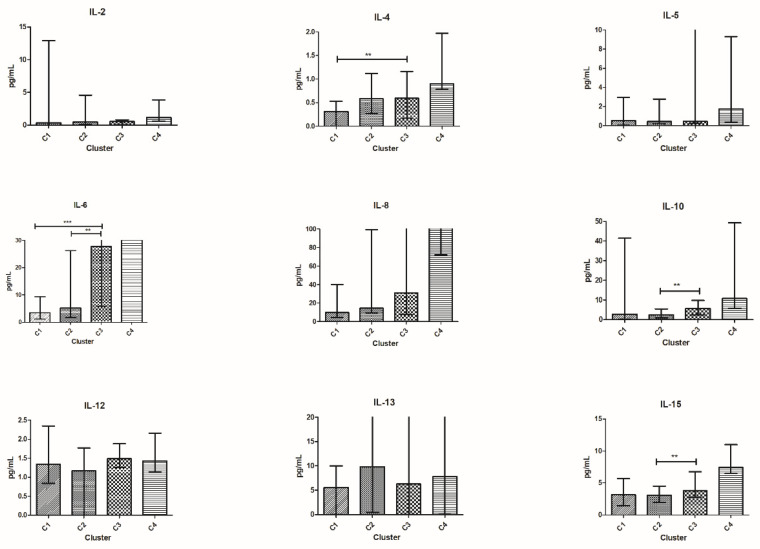

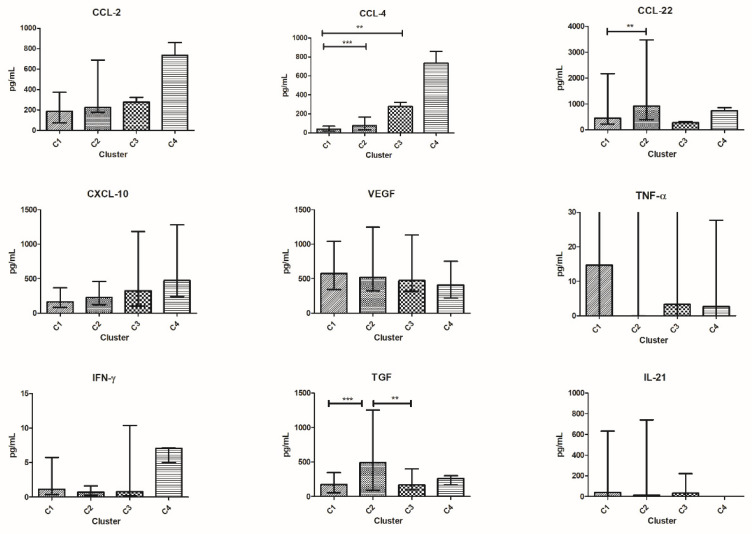
Cytokine distribution at T_1_ in the four clusters. Cytokine concentration in plasma is expressed in pg/mL and plotted on the *y*-axis; clusters of patients are on the *x*-axis. For TNF-α, IL-5, IL-6, IL-8, and IL-13 outliers are not shown for graphical choice. Data are expressed as median with range. *** *p* < 0.001, ** *p* < 0.01, * *p* < 0.05. CCL, C-C motif ligand; IL, interleukin.

Patients in C2^T1^ had lower median values of IL-6, IL-10, and IL-15 and higher median TGF-β values compared to those in C3^T1^ (Figure 7).

No clear differences between patients in C4^T1^ and each other cluster were observed in median cytokine values. However, C4^T1^ accounted for three patients only.

## 4. Discussion

This exploratory study focuses on cytokine expression and their modification during treatment with eribulin, and the correlation between them and patients’ outcomes. Albeit eribulin is a chemotherapy agent, it exerts some effects on circulating chemokines [8]. Therefore, eribulin allows the study of the correlation between cytokine modulation and patients’ outcomes.

We evaluated the differences among cytokines between patients before treatment (T0) and HV. Not surprisingly, we found that the median value of many cytokines was different in HV compared to patients. However, IL-2, IL-12, IL-15, TNF-α, (Th-1 cytokines), and CCL-22 did not change significantly between HV and patients. All these cytokines are linked to acute inflammation [17]. IFN-γ, differently from Th-1 cytokines, was higher in patients compared to HV. IFN-γ plays a crucial role in immune response. However, IFN-γ induces many pro-tumor reactions, including up-regulation of programmed death-ligand 1 (PD-L1), and Indoleamine 2,3-dioxygenase (IDO) affects T-cell immune response and, ultimately, has both pro- and antitumor properties depending on the concentration in the TME (reviewed in [25]). In line with the importance of pro-tumor effects of this cytokine, Jabeen et al. [26] observed reduction in IFN-γ in breast cancer patients responding to treatment with bevacizumab and chemotherapy.

The remaining cytokines, mostly significantly higher in patients, are related to Th-2 response and reflect the existing chronic inflammatory status [27].

After clustering patients through PCA and HCPC, we observed that the differences between HV and each cluster were not homogeneous. Lower levels of IL-6, IL-8, CCL-2, CXCL-10, and VEGF were observed when HV were compared to the patient clusters C2^T0^, C3^T0^, and C4^T0^. On the contrary, TGF-β level was significantly lower in HV compared to patients in C2^T0^ and C3^T0^, and IL-21 was lower compared to C3^T0^. Only two cytokines were higher in HV and limited to the comparison to C2^T0^ (IL-13) and C4^T0^ (TNF-α). These differences among HV and each specific patient cluster underline that the clusters identify different TME and suggest the existence of multiple different immune-escape mechanisms even in a small series of patients such as our population.

The comparison among clusters at T0 revealed that the median OS of patients in C2^T0^ was double compared to all other patients, with a 64% risk reduction in death. Patients in C2^T0^ are characterized by a lower median level of many important immunosuppressive cytokines, such as IL-6, IL-8, IL-10, and CCL-2. Of note, patients in C2^T0^ have better outcomes than patients in C3^T0^ showing the highest value of the same cytokines.

The analysis of the longitudinal shift among cytokines from T0 to T1 revealed significant changes only in patients clustered in C2^T0^ and C3^T0^. In particular, patients belonging to C2^T0^ showed an increase in IL-13 at T1, while patients in C3^T0^ exhibited IL-4 and VEGF increase. IL-2 grew at T1 in both clusters. IL-4 is reported as having an antiapoptotic effect [28] and its rise in C3^T0^ justifies the poor outcome of this cluster. Curiously, IL-13 is the only cytokine which increased from T0 to T1 in each patient in C2^T0^. Apart from IL-2 rising, which may indicate an initial recovery of immune response, the increase in IL-13 is intriguing. At T0, IL-13 is the only cytokine significantly higher in HV compared to patients in C2^T0^. IL-13 is historically related to the pathophysiology of asthma and other common autoimmune diseases [29] that may have a long pre-clinical stage [30]. It might explain the high variation of IL-13 observed in HV. IL-13, structurally similar to IL-4, plays a role in tumor proliferation and metastatization and is considered a Th-2-derived protein [31]. However, no clear relationship between IL-13 levels and poor outcome in breast cancer patients has been described [32]. IL-13 is involved in inhibition of inflammatory cytokines [33], up-regulation of tumor associated macrophages (TAM), and myeloid derived suppressor cells (MDSC) [34] without affecting activated T cells [35,36]. Therefore, the increase in IL-13 can be considered a signal of reactivation of the immune response as well. Indeed, in patients in C2^T0^, IL-13 at T1 is similar to the values observed in HV, and may contribute to the good outcomes of these patients.

After four courses of eribulin (T1), PCA and HCPC identified only four clusters. This may suggest that treatment reduces the heterogeneity of the TME.

Among them, patients in C1^T1^ showed the best outcome with a long median PFS and OS leading to a 50% and 63% risk reduction in disease progression and death, respectively.

PFS in the remaining clusters is very similar among them, while OS gradually decreases from C1^T1^ to C4^T1^ without overlapping the confidence intervals between C2^T1^ and C3^T1^.

Patients in C1^T1^ expressed very low median values of immune-suppressive cytokines, such as IL-4, IL-6, IL-8, CCL-4, and TGF-β. All of these are linked to major pro-tumor effects and poor survival [37,38,39,40]. Their low median values in C1^T1^ may represent a positive factor and may contribute to the best outcomes observed in this cluster.

Cytokine expression in the two best clusters, C1^T1^ and C2^T1^, is quite similar. However, significant differences exist in the median values of TGF-β, CCL-4, and CCL-22, that are higher in C2^T1^. TGF-β is a well-known negative prognostic factor, already demonstrated in many tumors [39,41]. CCL-4 and CCL-22 are also related to a poor prognosis [40,42] and may contribute to explaining the different outcomes of patients in C1^T1^ and C2^T1^. Intriguingly, TGF-β was higher in C2^T1^ than in C3^T1^, the latter showing a worst outcome compared to the former. However, many other Th-2 cytokines were higher in C3^T1^ than in C2^T1^. This observation stresses the concept that focusing on a single cytokine is not adequate, as cytokines interact with each other as previously reported [1].

Even the limited magnitude of the different OS between C1^T1^ and C2^T1^ underlines that many factors may contribute to, attenuate or exacerbate, the effects related to a single cytokine.

Therefore, clustering patients using a panel of multiple cytokines analyzed by PCA and HCPC may offer a better way to understand the TME and the complex interplay among its many components, such as tumor cells, stroma, and immune cells, and may explain the context dependent effects of many proteins [43].

It would be interesting to link our data with the molecular subtypes and metastatic profiles of the patients. For example, Kawaguchi et al. [18] demonstrated a different cytokine signature between metastatic and non-metastatic breast cancer patients. Unfortunately, due to the limited number of patients and the many variables to be considered, this analysis would not have had adequate statistical power.

We grouped our population into four groups according to PFS and OS. Patients of all groups were dispersed among the clusters either at T0 or T1. Only patients of group 2, showing short PFS and long OS, were mainly allocated in cluster C2^T0^. At T1, four of these patients were included in C1^T1^ and four in C2^T1^. Even if the former cluster showed the best behavior, the latter approached a C1^T1^ OS curve that is clearly separated from the OS curves of the two remaining clusters. Therefore, eight of nine patients included in group 2 are included in the clusters with the best OS.

All nine patients representing group 2 received further treatments after eribulin, but no one achieved an objective response. Therefore, the longer OS cannot be attributed to the further treatments. Indeed, Cox analysis, performed using confounding factors such as the number of lines of therapy, number of metastases, and different sites of deposits, confirmed that belonging to the C2^T0^, which encompasses seven out of nine patients of group 2, was an independent prognostic factor.

Interestingly, Haddad et al. reported that a subgroup of patients with head and neck cancer, treated with nivolumab in the CheckMate 141 study, benefited from continuing treatment beyond progression, on investigator choice, if they met specific clinical characteristics such as investigator-assessed clinical benefit, no rapid disease progression, treatment tolerance, and stable performance status [44]. Continuing treatment beyond progression converted initial progression to PR in almost 25% of these patients.

In our series, patients of group 2 met all these characteristics. Therefore, we might hypothesize that treating these patients beyond early progression could improve their outcomes. Indeed, our study suggests that the empirical clinical characteristics used by Haddad et al. might correspond to specific clusters of circulating cytokines.

## 5. Conclusions

We are aware that our study may only generate hypotheses for further investigations.

With this limitation, our data show the heterogeneity of the cancer-patient population even when we selected patients with the same tumor and clinical stage. However, considering circulating cytokines, we can group the population into six clusters with different outcomes. In addition, we can highlight that eribulin may reduce heterogeneity as witnessed by the decreased number of clusters after treatment.

Moreover, our data imply the possibility of using a low-invasive approach, such as serum analysis, to assess information regarding prognosis, and are supported by similar experiences from other authors [18,26,45]. This approach might be translated into clinical practice, in order to help physicians to identify cytokine clusters correlating to patients’ outcomes.

In our opinion, the most relevant hypothesis is that some patients, despite early progression, might benefit from treatment beyond progression. If this theory should be confirmed, this approach might be rapidly translated into clinical practice.

In support of the hypothesis, we refer to the Checkmate141 study conducted in patients with a different solid tumor and treated with different drugs.

However, when the target of treatment is TME, such as for immunotherapy or for the immune off-target effects of conventional chemotherapy, we face three major situations: inflamed tumors, excluded tumors, or desert tumors, which are the same across all solid tumors, with different distribution in different primaries [46]. Therefore, differences among primary sites may be much less relevant.

For these reasons, we believe that this hypothesis deserves further investigation.

## Figures and Tables

**Table 1:** Patients’ characteristics.

Characteristics	Number (%)
**Age (median, range)**	62 (37–86)
**ECOG PS (median, range)**	0 (0–2)
**De novo disease**	14 (34.1)
**ER status**	
Positive	34 (82.9)
Negative	7 (17.1)
**PgR status**	
Positive	28 (68.3)
Negative	13 (31.7)
**Triple negative**	6 (14.6)
**HER2 status**	
Positive	3 (7.3)
Negative	38 (92.7)
Neo/adjuvant CT (*)	20 (74.1)
Adjuvant ET (*)	22 (81.5)
**Number of previous CT lines for advanced disease**	
1	10 (24.4)
2	20 (48.8)
3	5 (12.2)
≥4	6 (14.6)
Median, range	2 (1–6)
**Number of previous ET lines for advanced disease**	
1	6 (14.6)
2	15 (36.6)
≥3	11 (26.8)
Median, range	2 (0–4)
**Number of organs involved**	
1	3 (7.3)
2	14 (34.1)
≥3	24 (58.5)
Median, range	3 (1–6)
**Most common metastatic sites**	
Bone	31 (75.6)
Liver	29 (70.7)
Soft tissue	27 (65.8)
Lung	17 (41.5)
Pleura	7 (17.1)
CNS	5 (12.2)
Peritoneum	4 (9.7)

(*) Numbers and percentages refer to 27 patients since 14 patients were metastatic at presentation. CNS, central nervous system; CT, chemotherapy; ECOG PS, Eastern Cooperative Oncology Group performance status; ER, estrogen receptor; PgR, progesterone receptor; ET, endocrine therapy; HER2, Human Epidermal Growth Factor Receptor 2.

**Table 2:** Group 2 patients’ characteristics.

Patient ID.	N. of Eribulin Cycles	ECOG PS at PD	Indolent Disease at PD	Symptoms at PD	CB	OS (mos)	N. of Previous ET + CT before Eribulin	Treatment at PD	Response to Treatment at PD
2	4	Stable	No	No	Yes	23.4	2 + 2	NPLD	SD
18	4	Stable	No	No	Yes	21.3	2 + 2	Nab-P	PD
22	4	Stable	No	No	Yes	11.5	3 + 2	mCAPE	PD
19	4	Stable	No	No	Yes	12.5	2 + 2	mVNR + CAPE	PD
8	4	Stable	No	No	Yes	14.8	2 + 2	Nab-P	SD
24	4	Stable	No	No	Yes	18.1	1 + 1	EVE-EXE	PD
27	4	Stable	No	No	Yes	11.0	2 + 1	Palbo + Letro	PD
34	4	Stable	No	No	Yes	20.4	0 + 2	CAPE	SD
43	4	Stable	No	No	Yes	13.4	3 + 1	PLD	SD

PD, progressive disease; CB, clinical benefit; SD, Stable Disease; ET, endocrine therapy; CT, chemotherapy; NPLD, non-pegylated liposomal doxorubicin; Nab-P, Nab-paclitaxel; mVNR, Metronomic Vinorelbine; mCAPE, Metronomic Capecitabine; Palbo, Palbociclib; Letro, Letrozole; EVE, Everolimus; EXE, Exemestane; PLD, pegylated liposomal doxorubicin.

**Table 3:** Cytokine comparison from T0 to T1 and from both timepoints compared to HV. Concentrations are expressed in pg/mL.

Patients (*n* = 41)			Patients (*n* = 41)			HV (*n* = 15)		
Cytokines at T0	Median (Range)	*p*-Value vs. T1	Cytokines at T1	Median (Range)	*p*-Value vs. HV	Cytokines HV	Median (Range)	*p*-Value vs. T0
IL-2	0.27 (0.00–3.17)	0.0004	IL-2	0.43 (0.00–12.90)	ns	IL-2	0.40 (0.00–0.80)	ns
IL-4	0.07 (0.00–2.82)	0.0001	IL-4	0.53 (0.00–1.97)	0.0003	IL-4	0.15 (0.08–0.68)	0.0045
IL-5	0.31 (0.00–20.2)	ns	IL-5	0.46 (0.06–14.60)	ns	IL-5	0.56 (0.04–5.24)	ns
IL-6	6.52 (0.82–212.4)	ns	IL-6	7.73 (1.15–216.1)	0.0001	IL-6	0.36 (0.00–3.70)	0.0001
IL-8	15.09 (0.57–542.4)	ns	IL-8	14.95 (3.17–240.6)	0.0001	IL-8	3.31 (2.02–5.76)	0.0001
IL-10	2.61 (0.37–28.7)	ns	IL-10	3.52 (0.16–49.26)	0.0001	IL-10	1.68 (0.90–2.56)	0.0062
IL-12	1.55 (0.0–3.17)	ns	IL-12	1.32 (0.00–2.35)	ns	IL-12	1.47 (0.47–70.36)	ns
Il-13	0.34 (0.00–1063)	0.0001	IL-13	6.17 (0.00–916)	ns	IL-13	8.14 (2.36–74.36)	0.0001
IL-15	3.23 (0.27–16.80)	ns	IL-15	3.27 (1.44–11.00)	ns	IL-15	3.10 (0.65–15.45)	ns
IL-21	32.53 (0.0–973.5)	ns	IL-21	22.04 (0.0–742.7)	0.0018	IL-21	0.00 (0.00–143.5)	0.0008
TGF-β	204.8 (86.55–915.9)	ns	TGF-β	233.9 (54.09–1255)	0.0001	TGF-β	90.24 (31.33–160.2)	0.0001
TNF-α	0.43 (0.0–737.7)	ns	TNF-α	2.65 (0.00–879.8)	ns	TNF-α	8.24 (2.50–50.00)	ns
VEGF	495.2 (224.9–1503)	0.0045	VEGF	523.7 (220.7–1248)	0.0001	VEGF	30.70 (9.80–186.3)	0.0001
IFN-γ	0.91 (0.03–7.79)	ns	IFN-γ	1.04 (0.20–10.40)	0.0039	IFN-γ	0.12 (0.00–2.10)	0.0091
CCL-2	231.4 (56.08–995.2)	ns	CCL-2	225.5 (74.09–878.1)	0.0001	CCL-2	44.72 (18.49–187.0)	0.0001
CCL-4	61.50 (0.00–125.8)	ns	CCL-4	62.73 (20.56–166.3)	0.0002	CCL-4	30.67 (4.27–67.20)	0.0003
CCL-22	497.4 (56.08–2727)	ns	CCL-22	476.2 (115.4–2173)	ns	CCL-22	347.2 (100.2–819.0)	ns
CXCL-10	210.1 (12.98–1039)	ns	CXCL-10	229.3 (82.88–1282)	0.0001	CXCL-10	71.70 (49.65–128.0)	0.0001

CCL, C-C motif ligand; IL, interleukin; ns, not significant.

## Data Availability

Data supporting results can be found at the ARCO Foundation laboratory at Santa Croce e Carle Teaching Hospital (Cuneo, Italy).

## Supplementary Materials

The following are available online at https://www.mdpi.com/article/10.3390/vaccines10010078/s1, Figure S1: Multivariate Cox analysis, Exp(B) = HR; Sign = *p*-value.
