# Supplementary material for: Circulating Cytokines in Metastatic Breast Cancer Patients Select Different Prognostic Groups and Patients Who Might Benefit from Treatment beyond Progression
Source: Vaccines (Basel). 2022 Jan 5;10(1):78. doi: 10.3390/vaccines10010078 (PMC8781714; doi:10.3390/vaccines10010078)
Supplement: Supplementary file 1 [file vaccines-10-00078-s001.zip › vaccines-1475066-supplementary.pdf]

vaccines

MDPI

Supplementary Materials

		frequency (N)	-1
C2_T0_comparison	Other pts	29	0
	C2_T0	12	1
group_lines_eri	0=II line	10	0
	1=> II line	31	1
metastasis_site	1=bone/soft tissue	6	1
	2=visceral	35	0
metastasis_number	0==< 2	17	1
	1=> 2	24	0

f. Variable (0,1). 0 is benchmark where HR was calculated.

Variables

							Exp(B) 95,0% C.I.	
	B	SE	Wald	gl	Sign.	Exp(B)	Lower	Upper
C2_T0_comparison	-1,136	0,439	6,707	1	0,010	0,321	0,136	0,759
group_lines_eri	-0,077	0,430	0,032	1	0,857	0,926	0,398	2,152
metastasis_number	-0,230	0,429	0,288	1	0,591	0,794	0,343	1,840
metastasis_site	-0,320	0,644	0,247	1	0,619	0,726	0,205	2,566

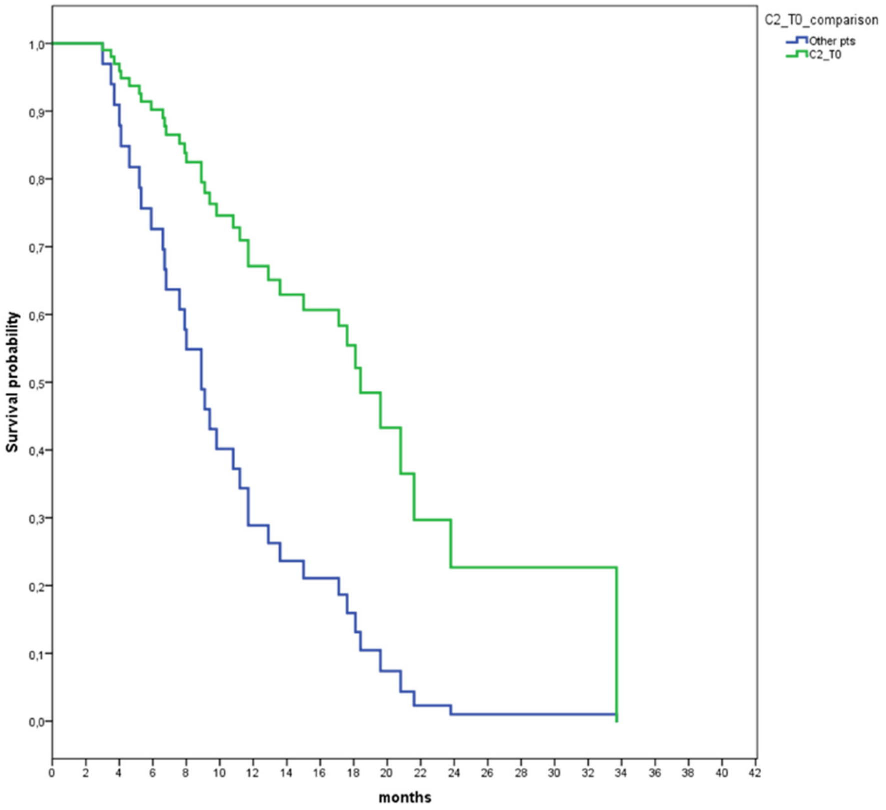

Figure S1. Multivariate Cox-analysis, Exp(B)=HR; Sign=p-value.
